# Targeting heparan sulfate proteoglycans as an effective strategy for inhibiting cancer cell migration and invasiveness compared to heparin

**DOI:** 10.3389/fcell.2024.1505680

**Published:** 2025-01-08

**Authors:** Lorenzo Depau, Jlenia Brunetti, Chiara Falciani, Elisabetta Mandarini, Marta Zanchi, Maria Francesca Paolocci, Marta Garfì, Alessandro Pini, Luisa Bracci

**Affiliations:** Department of Medical Biotechnologies, University of Siena, Siena, Italy

**Keywords:** heparan sulfate proteoglycans, cancer cell migration, peptide, extracellular matrix, tumor target

## Abstract

By virtue of their ability to bind different growth factors, morphogens and extracellular matrix proteins, heparan sulfate proteoglycans (HSPGs) play a determinant role in cancer cell differentiation and migration. Despite a strong conceptual basis and promising preclinical results, clinical trials have failed to demonstrate any significant advantage of administering heparin to oncology patients. We exploited our anti-heparan sulfate branched peptide NT4 to test the opposite approach, namely, targeting HSPGs to interfere with their functions, instead of using heparin as a soluble competitor in human cell lines from pancreas adenocarcinoma, colon adenocarcinoma, rhabdomyosarcoma and two different breast cancers. We found that the anti-heparan sulfate peptide NT4 is more effective than heparin for inhibiting cancer cell adhesion, directional migration, colony formation and even cell growth, suggesting that targeting cell membrane HSPGs may be a more effective anti-metastatic strategy than using soluble heparin. Analysis of NT4 effects on cancer cell directional migration, associated to cellular distribution of HSPGs and cadherins in different migrating cancer cell lines, provided further indications on the molecular basis of HSPG functions, which may explain the efficiency of the HSPG targeting peptide.

## Introduction

The development of metastases happens with most cancers and is the leading cause of cancer-associated death. In solid tumours, the transformation of cancer cells from an epithelial-like to a motile mesenchymal phenotype, known as the epithelial-mesenchymal transition (EMT), is the initiating event for cancer invasiveness. EMT is not exclusive to pathological conditions but also regulates tissue differentiation during morphogenesis and tissue regeneration in adults.

The cellular, biochemical and biophysical mechanisms regulating EMT and cell migration have been extensively studied because of their importance in physiological and pathological conditions. EMT-inducing ligands, such as many growth factors, TGF-beta, and the morphogenic ligands Wnt, Hedgehog and Notch all bind to heparin, heparan sulfate (HS) and heparan sulfate proteoglycans (HSPGs), which can act as co-receptors, although their precise role in signal transduction has not been fully clarified (Hassan et al., 2021; Marques et al., 2021; Soares da Costa et al., 2017; Hull et al., 2017).

HSPGs have a protein core carrying long linear chains of sulfated glycosaminoglycans (GAG) and can either be soluble in the extracellular matrix (ECM) or anchored to the cell membrane. Membrane-bound HSPGs can be divided into syndecans, which have a transmembrane protein core and intracellular domains, and glypican, which are anchored to the cell membrane through a glycosylphosphatidylinositol link (Hassan et al., 2021). GAG chains of HSPGs can vary greatly in terms of molecular weight (MW) and number and position of sulfated groups. In cancer cells, HSPGs and different enzymes involved in the synthesis and modification of GAG chains are often dysregulated, resulting in high variability in different cancers and in different stages of cancer progression (Marques et al., 2021; Soares da Costa et al., 2017). Adding to the complexity of their function, HSPGs and GAG chains can be released by cancer cells into the ECM through protease and endoglycosidase cleavage, and sulfation of GAG chains on the cell membrane and ECM can be modified by sulfatases (Hull et al., 2017).

The interaction of HSPGs with their ligands is mainly mediated by electrostatic bonds between multiple positively charged residues on the ligand and the negative charges of sulfate groups on the GAG chains (Vallet et al., 2021).

Thus, the number and position of sulfate groups can modulate ligand binding and HSPG biological function, playing a crucial role in different steps of cell differentiation and cancer progression (Poulain and Yost, 2015).

Although HSPGs have been shown to play a role in cell signalling in physiological conditions and in cancer (De Pasquale and Pavone, 2020), due to their high variability and the complex interplay between the numerous heparin-binding sites and ligands, the molecular basis of HSPGs' role in EMT and in cancer cell adhesion, migration and invasiveness is not fully understood. Nonetheless, drugs that can interfere with HSPG functions have been studied for decades for potential use in oncology (Onyeisi et al., 2020).

Heparin analogues have been investigated for their effectiveness in interfering with HSPG functions and have also been tested in cancer patients, not only for the treatment of cancer-associated thromboembolism but also for their potential effects on cancer growth and metastasis (Ma et al., 2020; Mohamed and Coombe, 2017). Despite promising preclinical and some clinical results (Ripsman et al., 2020) and contrary to previous reports suggesting the potential efficacy of heparins in cancer therapy, analysis of the data from numerous clinical trials involving the use of low molecular weight heparin has shown that there are no significant benefits in terms of tumour progression or overall survival, in different cancer types (Ek et al., 2018; Meyer et al., 2018; Montroy et al., 2020; O'Reilly et al., 2020). A comprehensive study using individual participant data meta-analysis, published in Lancet Haematology (Schünemann et al., 2020) and discussed in an accompanying commentary with the title “Heparins as cancer therapy: in theory, they should have worked” (Posch et al., 2020), showed no survival benefits in cancer patients treated with heparin.

Despite the numerous indications of HSPGs' role in cancer growth and metastasis, using heparin as a soluble decoy for the various HSPG ligands does not appear to be the most effective approach. Exploiting a specific peptide ligand that targets HSPG sulfated GAG chains, we tested an alternative approach, namely, interfering with HSPG functions by directly targeting HSPGs themselves, rather than using heparin as a soluble competitor.

The tetra-branched peptide NT4 has been shown to selectively bind to cells and tissues from different human solid cancers (Falciani et al., 2010a; Brunetti et al., 2015a). The selectivity towards cancer tissues was identified as due to NT4 binding to HSPG sulfated GAG chains (Falciani et al., 2013; Brunetti et al., 2016). Like other heparin-binding ligands such as Wnt, NT4 also binds to LRP receptors. NT4 binding to cancer cells and tissues is inhibited by heparin, heparan sulfate as well as by other heparin-binding ligands (Falciani et al., 2013). The positively charged residues in the NT4 sequence have been identified as regulators for binding to heparin and LRP receptors (Falciani et al., 2013).

We used NT4 to validate HSPGs as potential tumour-associated antigens and markers in various human solid tumours (Falciani et al., 2010a; Brunetti et al., 2015a), demonstrating that highly sulfated HSPGs are extremely overexpressed in different human solid tumours. The same NT4 peptide has also been conjugated to different tracers or drugs and tested as a tumour-targeting agent for cancer cell imaging and therapy, demonstrating its potential as a cancer theranostic agent (Falciani et al., 2007; Falciani et al., 2010b; Falciani et al., 2011; Brunetti et al., 2018; Brunetti et al., 2015b). After characterization of NT4 specificity for sulfated GAG (Brunetti et al., 2019), the unconjugated peptide was used as a specific tool for studying the role of HSPGs in cancer cell migration and invasiveness. The objective was to propose HSPGs not only as tumour markers but also as potential drug targets, to interfere with cancer invasiveness and metastatic potential.

NT4 binding to human cancer cell lines resulted in either inhibition or increase of oriented migration in PANC-1 human pancreas adenocarcinoma and TE671 human rhabdomyosarcoma cancer cells, respectively, indicating a crucial but diverse role of HSPGs in oriented cell migration in different cancer cells (Depau et al., 2020).

We compared here the effectiveness of HSPG targeting with heparin used as a general soluble competitor, by testing NT4 and heparin for their effect on adhesion of different cancer cell lines to ECM supports and on cell growth, migration and colony formation of the same cell lines.

NT4 inhibited adhesion and oriented migration and colony formation of all cancer cell lines except TE671, whereas heparin was minimally effective or ineffective in the same tests.

Our results provide additional information on the role of membrane HSPGs in cancer cell oriented migration and suggest that direct targeting of membrane HSPGs may result in a more effective pharmacological approach compared to the use of heparin as a soluble competitor, for interfering with HSPG activities.

## Materials and methods

### Materials

Human collagen IV (Sigma-Aldrich C6745); fibronectin from human fibroblasts (cellular fibronectin, Sigma-Aldrich F0556); fibronectin from human plasma (plasma fibronectin, Sigma-Aldrich F2006); heparin sodium salt from porcine intestinal mucosa (Sigma-Aldrich H3149); GRGDSP peptide (Sigma-Aldrich SCP0157); E− and N-cadherin antibodies (Cell Signalling Technology 14,472 and 13,116); anti E-cadherin for immunofluorescence analysis (Abcam ab40772); anti-Heparan Sulfate 10E4 Epitope (Amsbio 370,255–1); anti-Syndecan 4 antibody (Invitrogen PA5-95950); anti-GAPDH antibodies (Invitrogen AM4300); anti-rabbit IgG, HRP linked antibody (Cell Signalling Technology 7,074); Alexa Fluor 488 phalloidin (Invitrogen A12379); streptavidin-Atto 550 (Sigma-Aldrich 96,404); Streptavidin-Alexa 647 (Invitrogen S32357); goat anti-rabbit IgG conjugated with Alexa Fluor 546 (Molecular Probes A11010); goat anti-mouse IgM conjugated with Alexa Fluor 546 (Invitrogen A-21045); Pierce 16% formaldehyde (w/v) methanol-free (Thermo Scientific 28,908).

### Peptide synthesis

Peptide synthesis was performed with standard Fmoc chemistry as previously described (Brunetti et al., 2016).

All compounds are >95% pure by HPLC (Supplementary Figure S4).

In all experiments NT4 peptide was solubilized at 1 mg/mL in water.

### Cell lines

PANC-1 human pancreas adenocarcinoma, TE671 human rhabdomyosarcoma, HT-29 human colon adenocarcinoma, MCF-7 and MDA-MB 231 human breast adenocarcinoma were grown in their recommended medium DMEM supplemented with 10% fetal calf serum, 200 μg/mL glutamine, 100 µg/mL streptomycin, 60 μg/mL penicillin, and maintained at 37°C, 5% CO_2_. Cell lines were purchased from ATCC and cell profiling was analysed to authenticate human cell lines (BMR Genomics).

### Flow cytometry

PANC-1, TE671, HT29, MCF-7 and MDA-MB 231 cells were seeded 2 × 10^5^ cells/well in 96-well U-bottom plates and incubated with 500 nM (equivalent to 4 μg/mL) biotinylated NT4 with 20 μg/mL or 10 μg/mL heparin for 30 min at room temperature in PBS, 5 mM EDTA and 1% BSA. Cells were finally incubated with 1 μg/mL Streptavidin-FITC. 10000 events were acquired on Guava Easy Cyte cytometer (Luminex Corp., Austin, TX, United States) and analysed with FCS express 6 software.

### Cancer cell adhesion assay

Cancer cell adhesion assays were performed in 96-well flat-bottom plates coated with 20 μg/mL human collagen IV or with 10 μg/mL fibronectin from human fibroblast (cellular fibronectin) or fibronectin from human plasma (serum fibronectin) in PBS for 2 h at 37°C to polymerize. 1 × 10^5^ cells/well were plated for 30 min at 37°C with different concentrations of NT4 (from 1 μM to 10 μM), heparin (from 0.55 µM to 11 μM) or RGD peptide (from 0.3 μM to 340 μM) in their media. Cells were fixed with PBS - 4% PFA for 15 min at room temperature and stained with 0.1% crystal violet in 200 mM MES (2-(N-morpholino) ethanesulfonic acid) pH 6.0 for 1 h at room temperature. The cells were then solubilized with 10% acetic acid, the absorbance was measured at 595 nm using a microplate reader and analysed using GraphPad Prism 10.3.1 software.

### Wound healing

PANC-1 (3.1 × 10^4^), TE671 (5.2 × 10^4^), HT29 cells (7.7 × 10^4^), MCF-7 (10.5 × 10^4^) and MDA-MB 231 (4.4 × 10^4^) were seeded on each side of a culture insert for live cell analysis (Ibidi, Munich, Germany), which had previously been placed in a collagen IV pre-coated 24-well plate (20 μg/mL diluted in PBS for 2 h at 37°C). Plates were incubated at 37°C and 5% CO_2_ to allow cells to grow to confluence. The inserts were then removed with sterile tweezers and the cells were treated with 70 μg/mL (10 μM) NT4 peptide or 70 μg/mL heparin in complete medium. The cells were allowed to migrate in the incubator of a DMi8 (Leica Microsystems) microscope. The same instrument was used to take a picture at time zero and every 10 min until the gap was closed. The directionality, Euclidean distance and accumulated distance of migrating cancer cells were then analysed by time-lapse microscopy using ImageJ software and Chemotaxis and Migration Tool plug-in. For each cell line, the experiment was repeated twice at the same conditions and 15 individual cell tracks for each experiment were randomly selected and analysed (total n = 30 cells for each cell line).

### Western blot

Cells were seeded in six-well plates (3 × 10^6^ cells per well), previously coated with 20 μg/mL collagen IV in PBS, and maintained overnight in a CO_2_ incubator. Cells were lysed according to the antibody supplier’s instructions (Cell Signalling). 20 µL/lane total proteins were separated by 12% SDS-PAGE and transferred to a nitrocellulose membrane (GE healthcare). The membrane was blocked with 5% w/v non-fat dry milk in TBS containing 0.1% Tween20 for 1 h at room temperature and then incubated overnight at 4°C with specific anti-E, anti-N cadherin and anti-GAPDH antibodies diluted with 5% w/v BSA in TBS containing 0.1% Tween20. After washing with TBS containing 0.1% Tween20, the membrane was incubated with horseradish peroxidase-conjugated anti-rabbit IgG (Cell Signalling).

### Immunofluorescence

PANC-1 (3.1 × 10^4^), TE671 (5.2 × 10^4^), HT29 (7.7 × 10^4^), MCF-7 (10.5 × 10^4^) and MDA-MB 231 (4.4 × 10^4^) cells were seeded on each side of a culture insert for live cell analysis (Ibidi, Munich, Germany), which had previously been placed on collagen IV pre-coated glass slides (20 μg/mL for 2 h at 37°C in PBS). Plates were incubated at 37°C and 5% CO_2_ to allow cells to grow to confluence. The inserts were then removed with sterile tweezers, and the cells were allowed to migrate in the incubator for 3 h. The cells were then fixed with PBS-4% PFA methanol free (Thermo Scientific) for 10 min, permeabilized with PBS-0.25% Triton X-100 for 15 min (only for staining of E− and N-cadherin and Syndecan 4), saturated with PBS-5% bovine serum albumin (BSA) for 60 min and then incubated with Alexa Fluor 488 Phalloidin 1:40 in PBS-1% BSA for 30 min.

Cells were incubated either overnight at 4°C or for 2 h at room temperature with different primary antibodies: anti-N-cadherin (1:200 in PBS-1% BSA), anti-E-cadherin (1:500 in PBS-1% BSA), anti-heparan sulfate 10E4 Epitope (1:250 in PBS-1% BSA) or anti-Syndecan 4 (1:100 in PBS-1% BSA).

Cells were finally incubated for 2 h at room temperature with different secondary antibodies: goat anti-rabbit IgG conjugated with Alexa Fluor 546 1:1,000 in PBS-1% BSA for E− and N-cadherin and Syndecan 4 staining or goat anti-mouse IgM conjugated with Alexa Fluor 546 1:500 in PBS-1% BSA for heparan sulfate staining. For NT4 staining cells were instead incubated for 30 min with 2 µM NT4 labelled with biotin in PBS-1% BSA and then incubated for 30 min with Streptavidin-Atto 550 1:2000 in PBS-1% BSA (or Streptavidin-Alexa 647 1:2000 in PBS-1% BSA for co-localization experiments).

Samples, mounted using Fluoroshield with DAPI (Sigma Aldrich), were analysed by confocal laser microscope (Leica TCS SP8) with 364–495–550–556–650 nm excitation and 458–518–570–573–671 nm emission filters for DAPI, Alexa Fluor 488, Atto 550, Alexa Fluor 546 and Alexa Fluor 647 respectively. All images were processed using ImageJ software (NIH).

### Colony formation or clonogenic assays

Cells were seeded into 6-well plates, previously coated with 20 μg/mL collagen IV, and allowed to attach for 24 h. Cells were then incubated with NT4 peptide (70 μg/mL, equivalent to 10 µM) or heparin (70 μg/mL) for 6 days. After this period, cells were washed with phosphate buffered saline (PBS), fixed with PFA-4% in PBS and stained with 0.1% crystal violet solution. Images were acquired using confocal microscopy (Leica TCS SP8) and analysed using ImageJ software.

### Cell viability assay

Cells were seeded (12,000 cells/well for 24-h experiment or 4,000 cells/well for 6-day experiment) into 96-well plates, previously coated with 20 μg/mL collagen IV, and allowed to attach for 24 h. The cell viability before treatment (time zero) was calculated. Cells were then treated with 70 μg/mL (10 µM) NT4 peptide and with 70 μg/mL heparin for 24 h or 6 days. After this period, cells were treated with MTT [3-(4,5-dimethylthiazol-2-yl)-2,5-diphenyltetrazolium bromide] 5 mg/mL in PBS for 3 h, lysed with lysis buffer (45% DMF, 10% SDS, pH 4.5) and finally the absorbance of the samples was measured at 595 nm and 650 nm. Cell growth was calculated by subtracting cell viability at time zero from that obtained at the end of incubation.

### Statistical analysis

All experiments were repeated at least twice and the data was presented as mean ± SD. The significance of differences was analysed by the two-tailed Student’s t-test using GraphPad Prism 10.3.1 software; p values are reported in figure legends.

## Results

### Specific binding of NT4 to different human cancer cell lines

We tested the binding of NT4 and the inhibition of this binding by heparin, using flow cytometry in various human cancer cell lines, including PANC-1 (pancreas adenocarcinoma), TE671 (rhabdomyosarcoma), HT29 (colon adenocarcinoma), MCF-7 and MDA-MB 231 (two breast adenocarcinomas). Consistent with our previous findings with different human cell lines, NT4 bound to all the cancer cell lines, and this binding was inhibited by heparin (Depau et al., 2017; Bracci et al., 2018). In all cell lines, 90% inhibition of NT4 binding was achieved with 10 μg/mL heparin (Supplementary Figure 1).

### Effect of NT4, heparin and RGD peptide on adhesion of different human cancer cell lines to ECM proteins.

We compared the effects of the NT4 tetra branched peptide with those of heparin and the RGD peptide (GRGDSP) on adhesion of different human cancer cell lines (PANC-1, TE671, HT29, MCF-7, and MDA-MB 231) to collagen, cellular or plasma fibronectin and uncoated cell culture wells.

The RGD peptide is known to inhibit cancer cell adhesion through RGD-binding integrins (Ludwig et al., 2021). Inhibition of cancer cell adhesion by NT4 or heparin may suggest a role of HSPGs.

**FIGURE 1:**
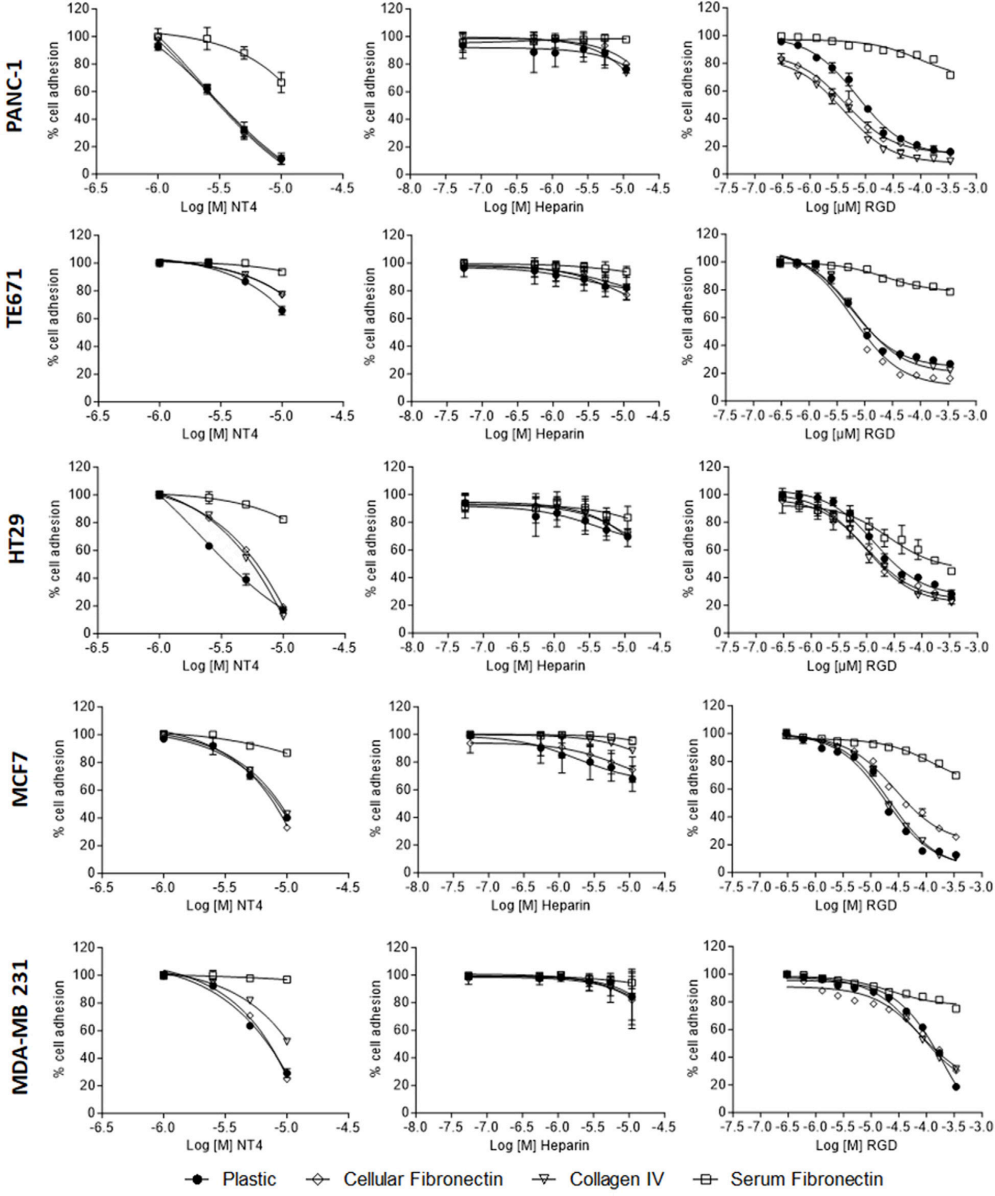
Adhesion of PANC-1, TE671, HT29, MCF7 and MDA-MB 231 human cancer cells to cellular fibronectin, plasma fibronectin, collagen IV and uncoated plates in the presence of NT4 (left), heparin (centre) and RGD peptide (right).

Consistent with our previous findings (Depau et al., 2020), we observed that NT4 inhibit adhesion of different cancer cell lines to collagen, cellular fibronectin and uncoated wells. However, the effect varied greatly in the different cell lines when tested in identical experiments, maximum inhibition reaching 90% in PANC-1 and HT29, and around 30% in TE671. NT4 had no effect on the adhesion of cancer cells to plasma fibronectin (Figure 1, graphs on the left).

The RGD peptide inhibited adhesion of all the cell lines to various supports, except plasma fibronectin. HT29 cells showed a unique pattern in which RGD inhibited adhesion to plasma and cellular fibronectin. Unlike for the NT4 peptide, the inhibition of adhesion by the RGD peptide was similar in all cell lines tested. RGD-mediated inhibition of cancer cell adhesion required higher peptide concentrations than that mediated by NT4 (Figure 1 graphs on the right).

Taken together, these results suggest that cancer cells can use HSPGs and integrins for adhesion to different ECM proteins. However, the role of integrins appears to be consistent across different cell lines, while the involvement of HSPGs in adhesion seems to depend more on the specific cell line. Interestingly, even at the highest concentrations, heparin was minimally effective in inhibiting adhesion of any cancer cell line to the different supports (Figure 1, graphs in the centre).

### Effect of NT4 and heparin on 2D migration of different human cancer cell lines

**FIGURE 2:**
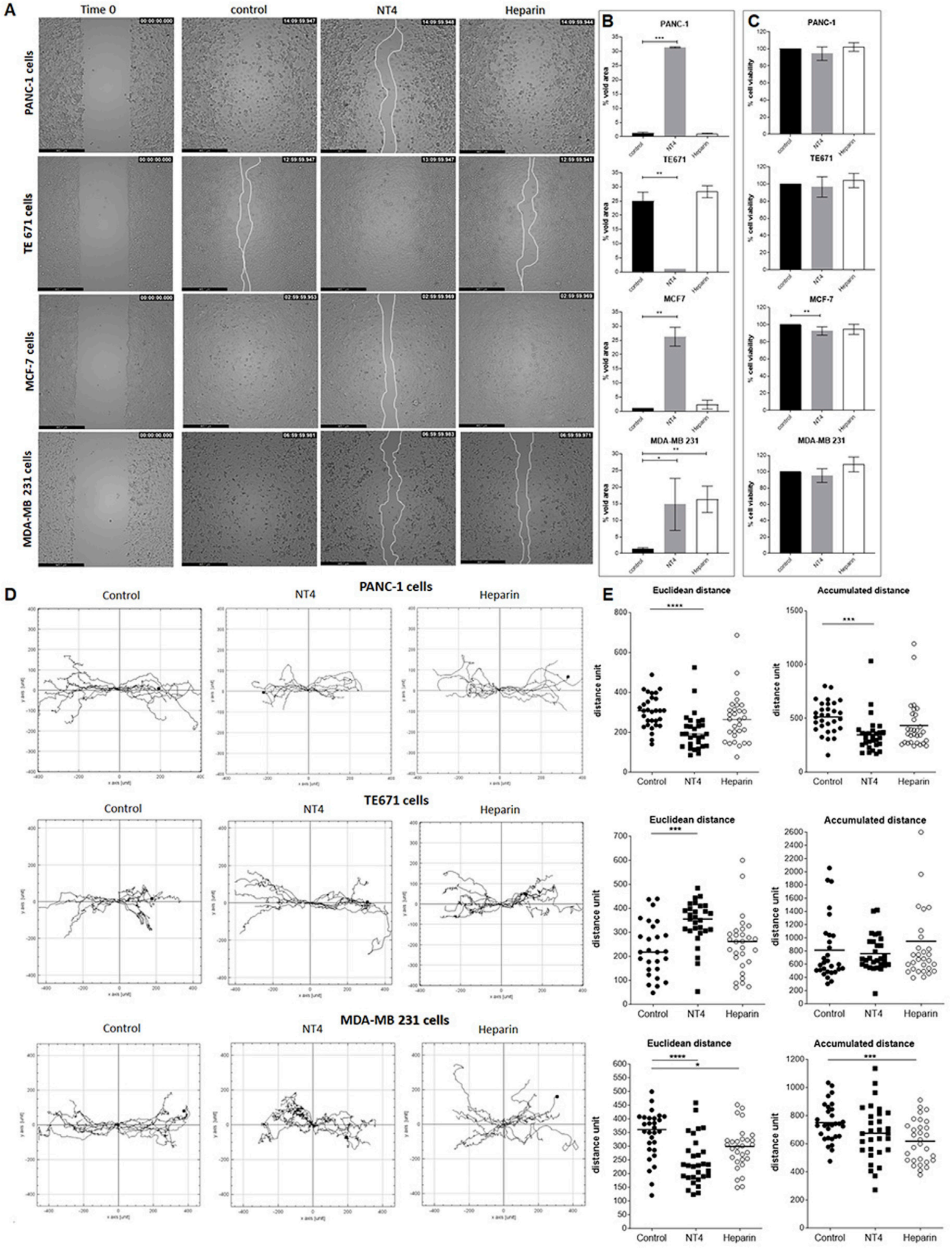
**(A)** Migration of cancer cells in wound-healing assays. PANC-1, TE671, MCF7 and MDA-MB 231 cancer cells were plated in wells coated with 20 μg/mL collagen IV. Once at confluence, cells were treated with 70 μg/mL NT4 peptide (10 μM) or with 70 μg/mL heparin and left until the void space was covered. Phase-contrast microscopy images were acquired immediately after removal of the spacer (time 0), and once the control or the treated well gap closed. **(B)** Void area at the end of the experiment was measured and expressed as a percentage of void space occupancy for different cancer cell lines, with or without treatment. **(C)** Effects of NT4 and heparin on cell viability, after 24 h of incubation. *p < 0.05, **p < 0.01, and ***p < 0.001 were calculated by the two-tailed Student’s t-test using GraphPad Prism 10 software. **(D)** Single cell tracking from time-lapse microscopy analysis. Phase-contrast microscopy images were acquired from each well from time zero and every 10 min until gap closure. Paths of single cells were monitored and plotted on a polar grid. Each grid represents 15 individual cell tracks, derived from a single experiment. **(E)** The Euclidean and accumulated distances were calculated from 30 individual cell tracks of two repeated experiments and reported in the box plot graphs. The median value is indicated by the black line. *p < 0.05, **p < 0.01, ***p < 0.001 and ****p < 0.0001 were calculated using the two-tailed Student’s t-test; n = 30.

The effects of peptide NT4 and heparin on the oriented migration of different cancer cell lines were tested using wound healing experiments in collagen IV-coated wells (Figure 2).

We had previously reported opposite effects of NT4 on the oriented migration of PANC-1 and TE671 cells on different ECM supports (Depau et al., 2020), confirming the crucial role of HSPG in cancer cell migration but also suggesting that this role can be different in different cancer cells.

With the aim of further analysing HSPG role in migration of different cancer cells, the study was here extended to different cancer cell lines. Moreover, to test whether HSPG targeting by NT4 can result in a more efficient interference with HSPG function, in the present study, we compared the effect of NT4 on the migration of PANC-1 and TE671 cells with that of heparin. The same analysis was extended to two different human breast cancer cell lines: MDA-MB231, a highly aggressive, invasive, poorly differentiated triple-negative cell line that displays single-cell migration, and MCF-7, a less aggressive cell line that retains several characteristics of differentiated mammary epithelium and migrates collectively. We found that NT4 inhibited migration of PANC-1 cells, as measured by void area percentage in the wound healing experiments, as we previously reported (Brunetti et al., 2016). However, heparin did not modify the migration of PANC-1 cells (Figures 2A,B). NT4 also significantly inhibited the migration of MCF-7 and MDA-MB231 cells. Heparin had no effect on the migration of MCF-7 cells, whereas it inhibited that of MDA-MB231 cells to a similar extent as NT4. In line with our previous findings, NT4 stimulated migration of TE671 cells, while heparin had no effect.

To examine the potential influence of NT4 and heparin on cell viability, which could confound the evaluation of cell migration based on void space occupancy, we incubated each cell line for 24 h with NT4 or heparin at the same concentrations used for the wound healing experiments and then stained viable cells with MTT. Cell viability was not significantly affected by NT4 or heparin in any cell line, except for low inhibition of cell growth of MCF-7 cells by NT4. However, inhibition of MCF-7 cell growth by NT4 was much lower than the inhibition produced by the same peptide on wound healing gap closure in the same cell line (Figure 2C).

We also analysed single-cell tracks from time-lapse videos of the wound healing experiments to assess directionality and velocity, obtaining valuable information on oriented migration of cancer cells beyond void space occupancy (Figure 2D).

For PANC-1, TE671, and MDA-MB 231 cells, single tracks of migrating cells in the presence of NT4 or heparin were analysed by measuring Euclidean distance (linear distance between start and end points) and accumulated distance (total length of each cell track) (Figure 2E). This analysis allowed us to differentiate the effect on orientation from that on velocity, both of which can affect gap closure. Since MCF-7 cells maintain cell-to-cell contacts and migrate collectively with a single migration front, analysis of single-cell tracks was not feasible (Supplementary Video 1).

In PANC-1 cells, NT4 significantly reduced the Euclidean and accumulated distances, whereas heparin had no significant effect.

In TE671 cells, NT4 significantly increased the Euclidean distance, whereas heparin had no significant effect. Neither NT4 nor heparin had a significant effect on accumulated distance in TE671 cells. These results confirm that the increase in cell migration, as measured by gap closure time, induced by NT4 in TE671 cells and not induced by heparin, was primarily generated by an increase in cancer cell directionality.

Among migrating cells, only MDA-MB 231 showed similar inhibition of cell migration by NT4 and heparin, in terms of gap closure. We obtained interesting findings from single cell tracking of migrating MDA-MB 231. NT4 significantly inhibited the Euclidean distance, which was also inhibited by heparin, albeit to a lesser extent. Accumulated distance was significantly inhibited by heparin, while NT4 had no significant effect. These results indicate that the inhibition of MDA-MB 231 migration by NT4 and heparin in wound healing experiments was generated by two different effects. NT4 inhibited cell directionality, whereas heparin mainly affected cell velocity.

### Location of HSPGs and cadherins in migrating cancer cells

Since results from HSPG targeting in wound healing experiments, suggested that HSPGs may have a role in guiding directional cell migration, cell distribution of HSPG and of cadherins, during oriented migration in wound healing experiments, was analysed by confocal microscopy.

**FIGURE 3:**
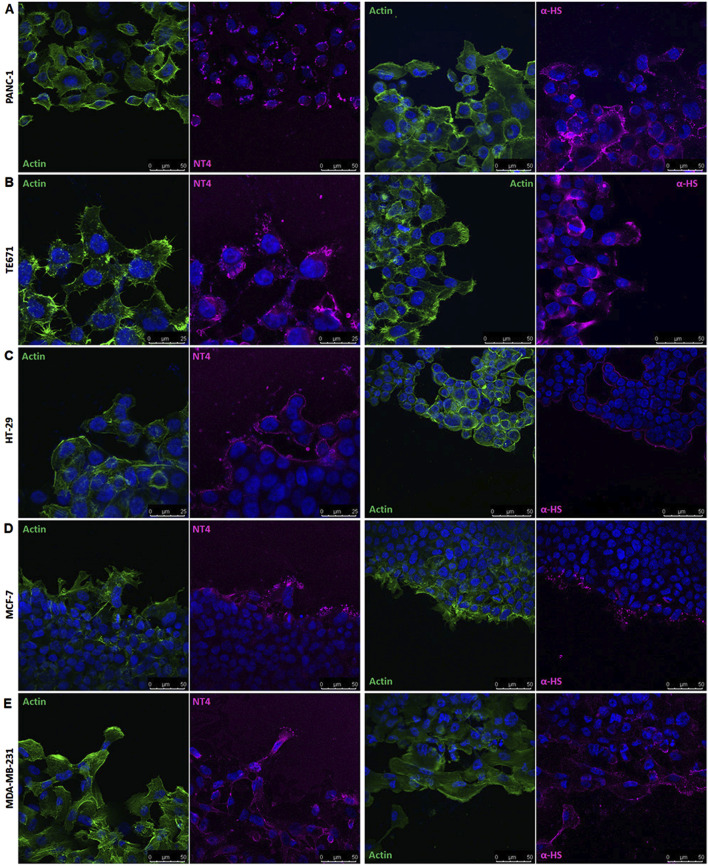
Localization of HSPGs in different human cancer cell lines under migrating conditions. Confocal microscopy analysis of NT4 peptide (magenta), anti-HS 10E4 antibody (magenta) and actin (green) in PANC-1 **(A)**, TE671 **(B)**, HT-29 **(C)**, MCF-7 **(D)** and MDA-MB 231 **(E)** cells. Nuclei are stained with 4′,6-diamidino-2-phenylindole (DAPI; blue).

**FIGURE 4:**
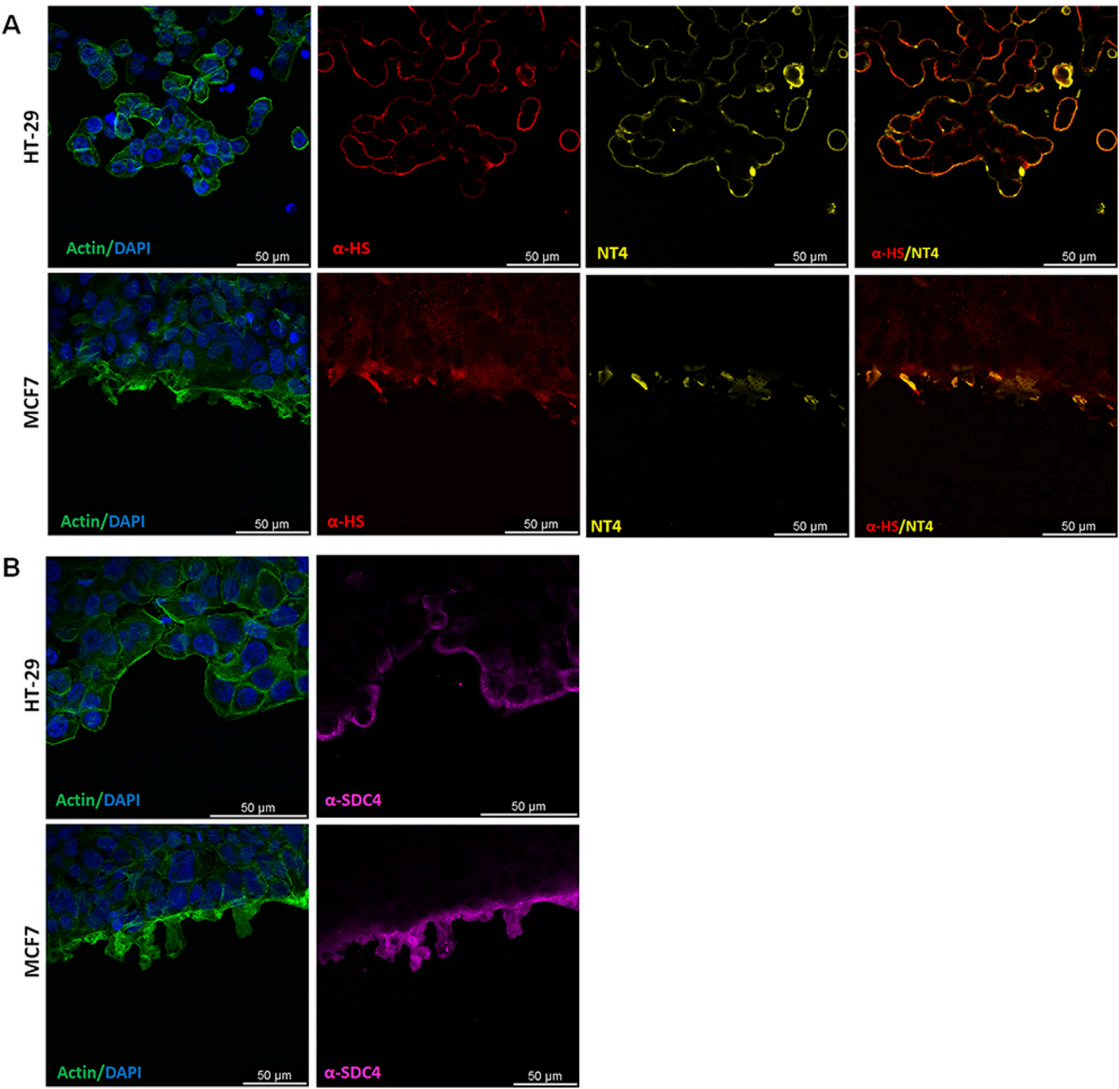
Localization of HSPG in HT29 and MCF-7 cell lines under migrating conditions. **(A)** Co-localization of anti-HS 10E4 antibody (red) and NT4 peptide (yellow) by confocal microscopy in HT29 and MCF-7 cells. **(B)** Confocal microscopy analysis of anti-syndecan4 antibody (magenta) and actin (green) in HT29 and MCF-7 cells. Nuclei are stained with 4′,6-diamidino-2-phenylindole (DAPI; blue) and actin is stained with phalloidin (green).

The binding of NT4 was compared to that of the anti-heparan sulfate antibody 10E4. As expected, the distribution of NT4 binding in migrating cells was similar to that of the anti-HS antibody in all cell lines (Figure 3 and Supplementary Figure 2). Interestingly, in cells migrating individually, such as PANC-1, TE671 and MDA-MB 231, HSPGs visualized by NT4 or 10E4 antibody, were distributed on the cell membrane without any clear polarization (Figures 3A, B, E and Supplementary Figure 2). However, in collectively migrating cells like MCF-7, HSPGs, were predominantly located at the leading edge of the migration front of leader cells (Figures 3D, 4). Surprisingly, a similar distribution of HSPGs was observed in HT29 cells, which do not migrate in wound healing experiments (Depau et al., 2020). Despite the inability of HT29 cells to migrate, HSPGs were selectively located at the cell front exposed to the void space (Figure 3C). The selective distribution of HSPG in MCF-7 and HT29 cells was clearly confirmed by confocal microscopy colocalization of signals produced by NT4 and 10E4 antibody binding (Figure 4A).

Since both NT4 and 10E4 antibody recognize HS chains, in order to verify whether the selective location of HSPGs on the migration front of MCF-7 and on the void space front of HT29 was due to a selective distribution of HSPGs or to an overproduction or over-sulphation of GAGs at the same front, we checked the distribution of HSPG protein core in the same migrating cells. We used an anti-syndecan 4 antibody, since we had found that this is expressed at a much higher level compared to other syndecans and glypicans, in all the cell lines we have here analysed, with the sole exception of TE671 (Brunetti et al., 2019; Depau et al., 2020). We found that distribution of syndecan 4 in MCF-7 and HT29 was identical to that of HSPGs as located by either NT4 or anti-HS 10E4 antibody, demonstrating that both the collectively migrating MCF-7 and the not migrating HT29 cells clearly concentrate syndecan 4 at the void space front (Figure 4B).

In a previous paper, we reported the expression and cell distribution of E− and N-cadherin in PANC-1, TE671, and HT29 cells. PANC-1 cells express E− and N-cadherin. In wound healing experiments, both cadherins were downregulated in PANC-1 cells at the migration front and were less visible in isolated migrating cells. TE671 cells express only N-cadherin, which was also downregulated in cells at the migration front and scarcely detectable in isolated cells. HT-29 cells express only E-cadherin, which was not downregulated and remained expressed around the cells, inside and at the migration front (Depau et al., 2020).

**FIGURE 5:**
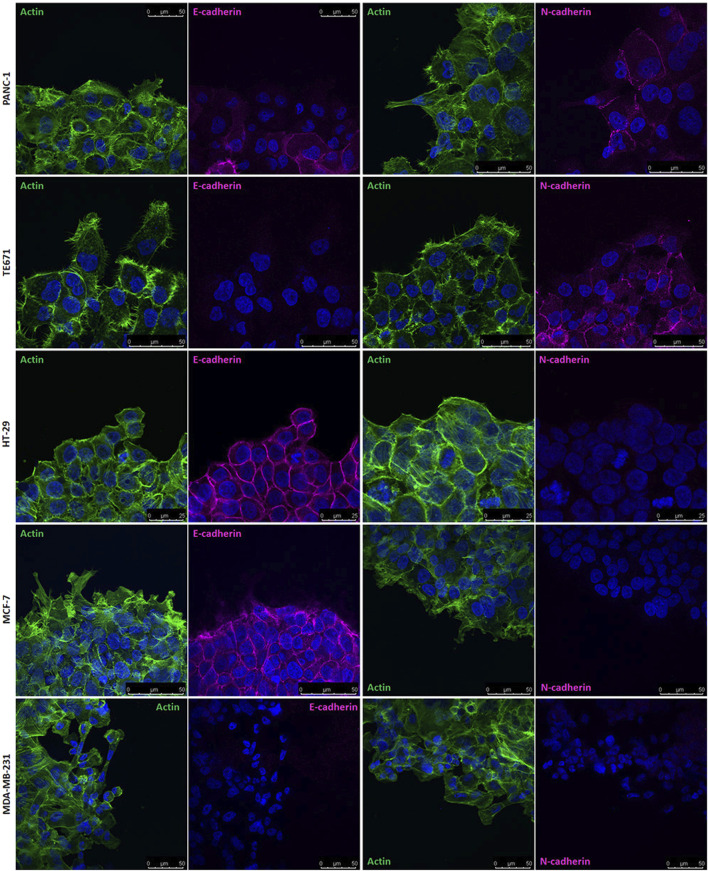
Localization of E− and N-cadherin in different human cancer cell lines under migrating conditions. Confocal microscopy analysis of E− and N-cadherin (magenta) and actin (green) in PANC-1, TE671, HT29, MCF-7 and MDA-MB 231 cells. Nuclei are stained with 4′,6-diamidino-2-phenylindole (DAPI; blue).

Based on these results, we examined the expression of E− and N-cadherin in MCF-7 and MDA-MB 231 cells using Western blot. We found that MCF-7 cells express only E-cadherin, while MDA-MB 231 cells have no detectable expression of either E− or N-cadherin (Supplementary Figure 3). We then located E-cadherin by confocal microscopy in MCF-7 cells migrating in wound healing experiments. We observed that even in MCF-7 cells, E-cadherin is downregulated at the migration front. Since MCF-7 cells migrate collectively, leader cells at the migration front maintain close contacts with follower cells. E-cadherin was clearly visualized along the cell contacts but was less expressed on the edge exposed to the migration front of leader cells. Confocal microscopy analysis of MDA-MB 231 cells confirmed the absence of E− or N-cadherin expression, as no cadherin was visualized using specific antibodies (Figure 5).

By comparing the cell distribution of cadherins and HSPGs as visualized by confocal microscopy in migrating cells in wound healing experiments, a general complementary distribution can be observed. E− and N-cadherin are concentrated at cell contacts and are generally barely detectable in isolated migrating cells. In contrast, HSPGs are expressed on the entire membrane of isolated migrating cells. This complementarity is particularly evident in collectively migrating MCF-7 cells, where HSPGs are exclusively located on the projections of leader cells at the front of the collectively migrating cell group, where E-cadherin is not visualized. Conversely, no evident expression of HSPGs is detected in the interior of the migrating cell group, where E-cadherin is highly expressed.

### Effect of NT4 and heparin on cancer cell colony formation

**FIGURE 6:**
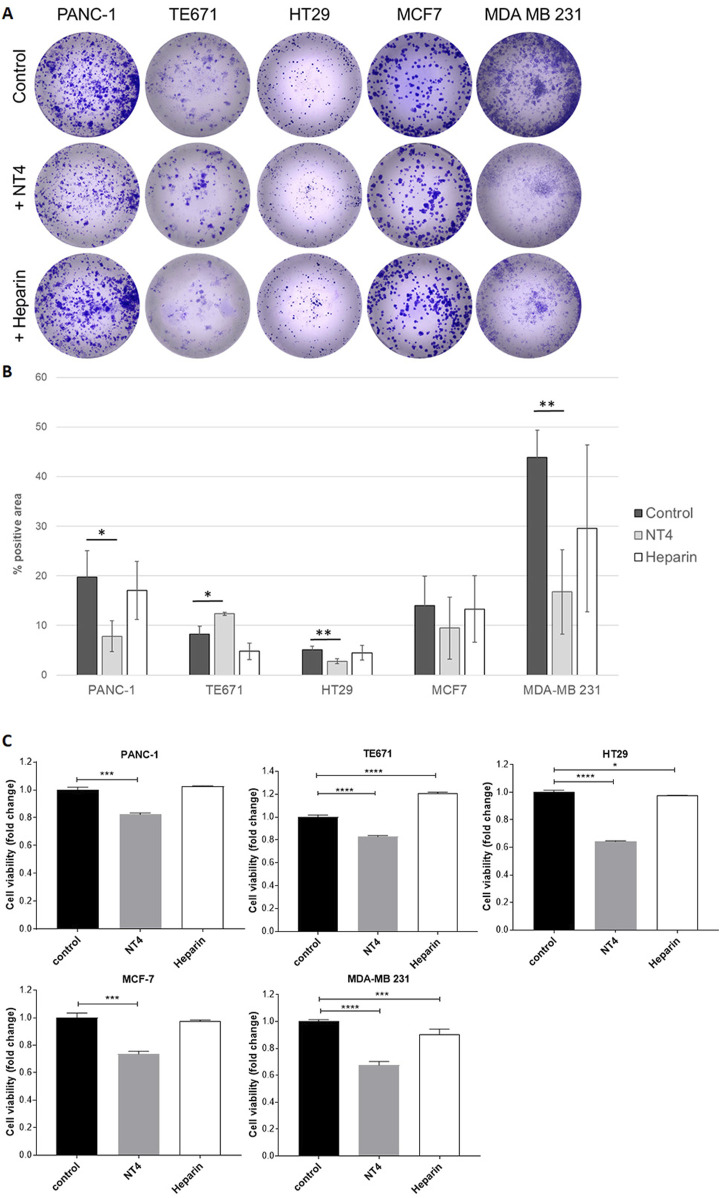
Effect of NT4 (70 μg/mL, equivalent to 10 μM) and heparin (70 μg/mL) on colony formation of different cancer cell lines. Highly diluted cells were seeded in collagen coated wells and incubated for 6 days. Colonies were then visualized by crystal violet staining **(A)**. Data is reported as the percentage of positive area calculated with ImageJ software **(B)**. The histograms show the effect of NT4 and heparin on cell viability of the different cell lines after 6 days of incubation **(C)**. *p < 0.05, **p < 0.01, ***p < 0.001 and ****p < 0.0001 were calculated by the two-tailed Student’s t-test using GraphPad Prism 10 software.

The colony formation assay is a valuable method for assessing the tumour-initiating capacity of cancer cells. It measures the ability of highly diluted cells to divide and give rise to colonies. We seeded diluted cancer cells in collagen-coated wells and incubated them for 6 days, after which the colonies were visualized and measured by staining techniques. Different cell lines showed varying potential for colony formation, resulting in colonies with distinct numbers and shapes (Figure 6).

NT4 inhibited colony formation in PANC-1 cells, and even more so in MDA-MB 231 cells. It did not significantly alter colony formation in MCF-7 cells. Although HT29 cells showed low colony formation, NT4 also induced significant inhibition in this cell line. Interestingly, NT4 led to a significant increase in colony formation in TE671 cells, which aligns with the observed stimulatory effect of NT4 on the oriented migration and *in vitro* invasiveness (Depau et al., 2020) of these cells. On the other hand, heparin did not exert a significant effect on colony formation in any of the cell lines.

Neither NT4 nor heparin affected cancer cell growth in the cell lines during a 24-h incubation period. This observation allowed us to assess that inhibition or stimulation of cell migration induced by NT4 or heparin in wound healing experiments was not influenced by alterations in cell growth. To account for potential effects of NT4 or heparin on cell viability, which may influence colony formation results, longer incubation times were examined. Notably, when the incubation time was extended to 6 days, NT4 produced a significant inhibition of cell growth in all cell lines tested, including TE671 cells, where the peptide resulted in a significant increase in colony formation in the same time frame. Intriguingly, heparin produced a significant increase in cancer cell growth under the same experimental conditions in TE671 cells. In contrast, heparin inhibited cell growth in MDA-MB 231 cells, although the inhibition was much less pronounced than that induced by NT4 in the same cells.

## Discussion and conclusions

Heparan Sulfate Proteoglycans are crucial in various physiological and pathological processes, including embryonic development, tissue regeneration and cancer. Since HSPGs are involved in cancer cell adhesion to the extracellular matrix and in cancer cell migration, drugs that can interfere with HSPG functions have been explored as potential therapeutic agents for cancer progression and metastasis.

In this context, heparin, and its low molecular weight analogues, acting as direct competitors of HSPGs, have been tested as inhibitors of HSPG activity in cancer through, the application of heparins in oncology has not consistently yielded positive outcomes in clinical trials (Ek et al., 2018; Meyer et al., 2018; Montroy et al., 2020; O'Reilly et al., 2020; Wieboldt and Läubli, 2022).

Preliminary findings indicated that the HS-binding peptide NT4 interfere with adherence and migration of human pancreatic cancer cells. We speculated that a different approach targeting cell membrane HSPGs to disrupt their function, might be more effective than using heparin as a soluble competitor of HSPGs for binding their numerous ligands. To investigate this, NT4 and heparin were compared for their impact on adhesion, migration and colony formation across various human cancer cell lines.

We compared the effects of NT4, heparin and the integrin-binding RGD peptide on the adhesion of different human cancer cell lines to ECM supports. Our results demonstrate that NT4 and the RGD peptide can both inhibit adhesion of cancer cells to various supports, while heparin cannot.

A possible explanation of the scarce inhibitory effect of heparin, may be the redundancy of heparin binding sites on ECM proteins, which may necessitate very high concentrations of heparin to achieve complete saturation or blocking. Given the abundance of heparin-binding site in the tumour microenvironment, this may partly explain the limited efficacy of heparin in interfering with cancer cell spread *in vivo*.

Adhesion to plasma fibronectin was not affected by NT4 and RGD in all cancer cell lines, except HT29. Possibly, cancer cells interact with plasma fibronectin with a mechanism that does not involve RGD-binding integrins or HSPGs. In contrast, integrins and HSPGs both seem to be involved in cancer cell binding to cellular fibronectin, which is a major component of the ECM and the tumour microenvironment.

The effect of NT4 peptide and heparin on the migration of different cancer cell lines was analysed in wound healing experiments. Consistent with our previous findings (Depau et al., 2020), NT4 inhibited the migration of PANC-1 cells and stimulated the migration of TE671 cells. Heparin did not have significant effects on the migration of either PANC-1 or TE671 cells under the same experimental conditions.

NT4 significantly inhibited migration of MCF-7 and MDA-MB 231 breast cancer cell lines, higher inhibition being observed in MCF-7 than MDA-MB 231 cells. Heparin had no effect on the migration of MCF-7 cells, whereas it produced inhibition comparable to NT4 on the migration of MDA-MB 231 cells.

Besides observing void space occupancy, we analysed single cell tracks from time lapse videos. Measurement of the Euclidean and accumulated distances of cell tracks revealed that NT4 primarily affected cell directionality. Analysis of single cell tracks was not feasible for MCF-7 cells since they migrate collectively.

The results from our analysis of cell migration confirm the crucial role of HSPGs, particularly in controlling cell orientation. In all the cancer cell lines tested, blocking cell membrane HSPGs with the HS-binding peptide NT4 affected the directionality of cell migration. This resulted in inhibition of migration in three out of 4 cell lines and stimulation of migration in TE671 cells. On the other hand, heparin generally had limited and non-significant effects on cell migration in all cell lines except MDA-MB 231, where it influenced accumulated distance rather than directionality.

The diverse effects of HS targeting in different cancer cell lines may be associated with expression of different syndecans and glypican, as we previously reported (Brunetti et al., 2019; Depau et al., 2020). Glypicans, which lack endocellular protein domains, may primarily be involved in the co-receptor activity of HSPGs, while syndecans, through their endocellular domains, may also be directly involved in various endocellular signalling pathways related to cell migration. In particular, syndecan 4 has been shown to be linked to cell polarization and directional migration (Keller-Pinter et al., 2021), through Rac1 regulation (Bass et al., 2007). Notably, syndecan 4 is highly expressed in PANC-1, MDA-MB 231 and MCF-7 cells, the cell lines whose migration and directionality were inhibited by NT4. In contrast, TE671 cells, the only cells among those tested here for which NT4 binding resulted in stimulation of cell migration, exhibited low expression of syndecan 4 and high expression of syndecans 2 and 3 (Brunetti et al., 2019; Depau et al., 2020).

Heparin, which does not bind to membrane HSPGs, cannot directly influence HSPG-mediated signalling. However, heparin can bind heparin-binding sites on ECM proteins and potentially affect cell velocity, provided its concentration is high enough to significantly inhibit HSPG binding to the same ECM binding sites, which may be difficult to achieve *in vivo*.

Further indications on the role of HSPG in directional cell migration came from the confocal localization of HSPGs and cadherins in migrating cancer cells. HSPG GAG chains were visualized employing biotin-conjugated NT4 or the anti-HS antibody 10E4, and compared with the distribution of cadherins, which are known to be crucial regulators of cell adhesion and migration (Grimaldi et al., 2020; Bischoff et al., 2021). Interestingly, HSPGs were uniformly distributed on the membrane of cancer cells that migrate individually. In the same cells, E or N-cadherins were generally evident at cell contacts, while they were downregulated in isolated migrating cells.

MCF-7 and MDA-MB 231 human breast cancer cell lines exhibit numerous morphological, physiological and molecular differences. MDA-MB 231 cells are highly invasive and multidrug resistant, characterized by a typical mesenchymal fibroblast-like morphology and single cell migration. On the other hand, MCF-7 cells have a distinct, more differentiated epithelial-like morphology and exhibit collective migration. Additionally, MCF-7 and MDA-MB 231 cells differ in their expression of E− and N-cadherin, which are known to be crucial regulators of cell adhesion and migration. MCF-7 cells express E-cadherin but not N-cadherin, whereas MDA-MB 231 cells do not express either.

In collectively migrating MCF-7 cells, HSPGs localized on the membrane of leader cells, specifically on the edge exposed to the void space, while the distribution of E-cadherin was the opposite to that of HSPGs, being almost undetectable at the front edge of the cells. This distribution of HSPGs at the front of migrating cells further supports the hypothesis of their fundamental role in the regulation of oriented cell migration.

In HT29 cells, which express E-cadherin and do not migrate in wound healing experiments, HSPGs were visualized on cell membrane along the border exposed to the void space, like in MCF-7. Interestingly, differently to what visualized in MCF-7, E-cadherin and HSPG in HT29 do not display a complementary distribution, and E-cadherin was well evident on the cell front exposed to the void space.

Since enzymes involved in GAG synthesis and sulfation are often misregulated in cancer cells, in order to verify whether the accumulation of HSPG on the cell membrane exposed to the void space in MCF-7 and HT29 was generated by a selective localization of HSPGs or by increased sulphated GAGs, we used an antibody recognizing the protein core of syndecan 4, which is the most expressed syndecan in both MCF-7 and HT29 cells (Brunetti et al., 2019; Depau et al., 2020). Results from confocal microscopy clearly indicate that syndecan 4 is selectively located on the cell membrane at the front exposed to the void space.

Extensive studies have explored the role of E− and N-cadherin on oriented cell migration, several reports indicating the essential role of cadherin-mediated cell-cell contacts in regulating the orientation of cell migration (Grimaldi et al., 2020; Bischoff et al., 2021). The complementary distribution of E-cadherin and HSPGs suggests that at least in collective migration, E-cadherin and HSPGs may collaborate in determining cell polarization and the coordination and orientation of cell migration. The distribution of E-cadherin and HSPG in HT29, which do not downregulate E-cadherin on the front and do not migrate in wound healing experiments, seems to confirm this hypothesis.

Furthermore, the effects observed with the sulfated-GAG-specific NT4 peptide on the directionality of migration in other cancer cells further confirm the determinant role of HSPGs in guiding directional cell migration.

Considering the already suggested role of syndecan 4 in oriented cell migration (Keller-Pinter et al., 2021; Bass et al., 2007), the clear and selective localization of syndecan-4 at the front of collectively migrating cells strongly suggest that syndecan 4 may be the pivotal component responsible for initiating and controlling cell signalling for collective migration of MCF-7 breast cancer cells.

NT4 and heparin were also compared by assessing their effects on colony formation in the same cell lines analysed in the wound healing experiments. The ability of cancer cells to form colonies at a very high dilution is considered representative of tumor initiating capacity of cancer cells, which together with oriented migration can provide indications on metastatic potential of different cancer cells. Number and form of colonies varied significantly among the tested cell lines. Heparin did not significantly modify colony formation in any cell line. NT4 inhibited colony formation in PANC-1 cells and slightly stimulated colony formation in TE671 cells, consistent with its effect on the migration of these cells. Colony formation in MDA-MB 231 cells was strongly inhibited by NT4.

NT4 and heparin did not show significant effect on the growth of any of the cancer cell lines when incubated for up to 24 h. Interestingly, after incubation for 6 days, the growth of all cell lines, including TE671 was significantly inhibited by NT4. Intriguingly, heparin significantly stimulated the growth of TE671 under the same experimental conditions, while it slightly inhibited the growth of HT29 and MDA-MB 231 cells. These results suggest that the colony stimulation produced by NT4 in TE671 cells is primarily generated by the increase in cell migration induced by NT4. On the other hand, the inhibition of colony formation in the other cell lines may be a result of the combined effects on cell migration and cell growth, both of which are inhibited by NT4, whereas heparin has little or no effect.

The inhibition of cell growth by NT4 on all cell lines during long incubation may be related to a possible interference with growth factor or morphogen-induced cell growth, which may be less evident in a 24-hour interval. In fact, by binding to cell membrane HSPGs, NT4 may interfere with all HSPG functions, including their known co-receptor role for many growth factors and morphogens.

The dramatic inhibition of colony formation produced by NT4 on MDA-MB 231 cells may be attributed to the combination of an NT4 effect on cell signalling directly associated with HSPGs, such as the regulation of oriented cell migration, with long-term inhibition of cell growth, which may be linked to the co-receptor role of HSPGs in growth factor signalling.

The possible interference of heparin in cell signalling associated with heparin-binding ligands may be very different to that of the HS-binding NT4 peptide. In fact, by binding soluble growth factors and presenting them to membrane receptors, heparin may even increase the local concentration of the ligands and subsequently activate the receptors. Indeed, heparin resulted in an increase in cell growth in TE671 cancer cells, whereas the anti-HS NT4 peptide clearly inhibited their cell growth under identical experimental conditions.

In conclusion, we found that targeting HSPGs with the anti-HS peptide NT4 is more efficient than using soluble heparin to interfere with HSPG role in *in-vitro* cancer cell adhesion, migration, invasiveness, and growth. Our results suggest that targeting cell membrane HSPGs can be an anti-metastatic strategy that may be more effective than using soluble heparin. However, a personalized, cancer cell-dependent approach is necessary, as HSPGs appear to have a crucial but diverse role in oriented migration of different cancer cells. Although we demonstrated that NT4 can inhibit cell growth in all the present cancer cell lines over time intervals longer than 24 h, and can also inhibit directional migration and colony formation in most of said human cancer cells, the increase in oriented migration and colony formation produced by the same peptide in TE671 indicates that targeting HSPGs with specific ligands may in some cases result in a pro-metastatic effect. Nonetheless, an analogous cancer cell-dependent strategy should also be considered for heparin therapies, as heparin clearly stimulates cell growth in TE671, while NT4 inhibits it.

## Abbreviation

EMT, epithelial-mesenchymal transition; HSPGs, heparan sulfate proteoglycans; GAG, sulphated glycosaminoglycans; ECM, extracellular matrix; HS, heparan sulfate.

## Data Availability

The raw data supporting the conclusions of this article will be made available by the authors, without undue reservation.

## Abbreviations

EMT, epithelial-mesenchymal transition; HSPGs, heparan sulfate proteoglycans; GAG, sulphated glycosaminoglycans; ECM, extracellular matrix; HS, heparan sulfate.
