# Supplementary material for: Targeting heparan sulfate proteoglycans as an effective strategy for inhibiting cancer cell migration and invasiveness compared to heparin
Source: Front Cell Dev Biol. 2025 Jan 8;12:1505680. doi: 10.3389/fcell.2024.1505680 (PMC11750806; doi:10.3389/fcell.2024.1505680)
Supplement: Supplementary file 1 [file DataSheet1.pdf]

## Supplementary material

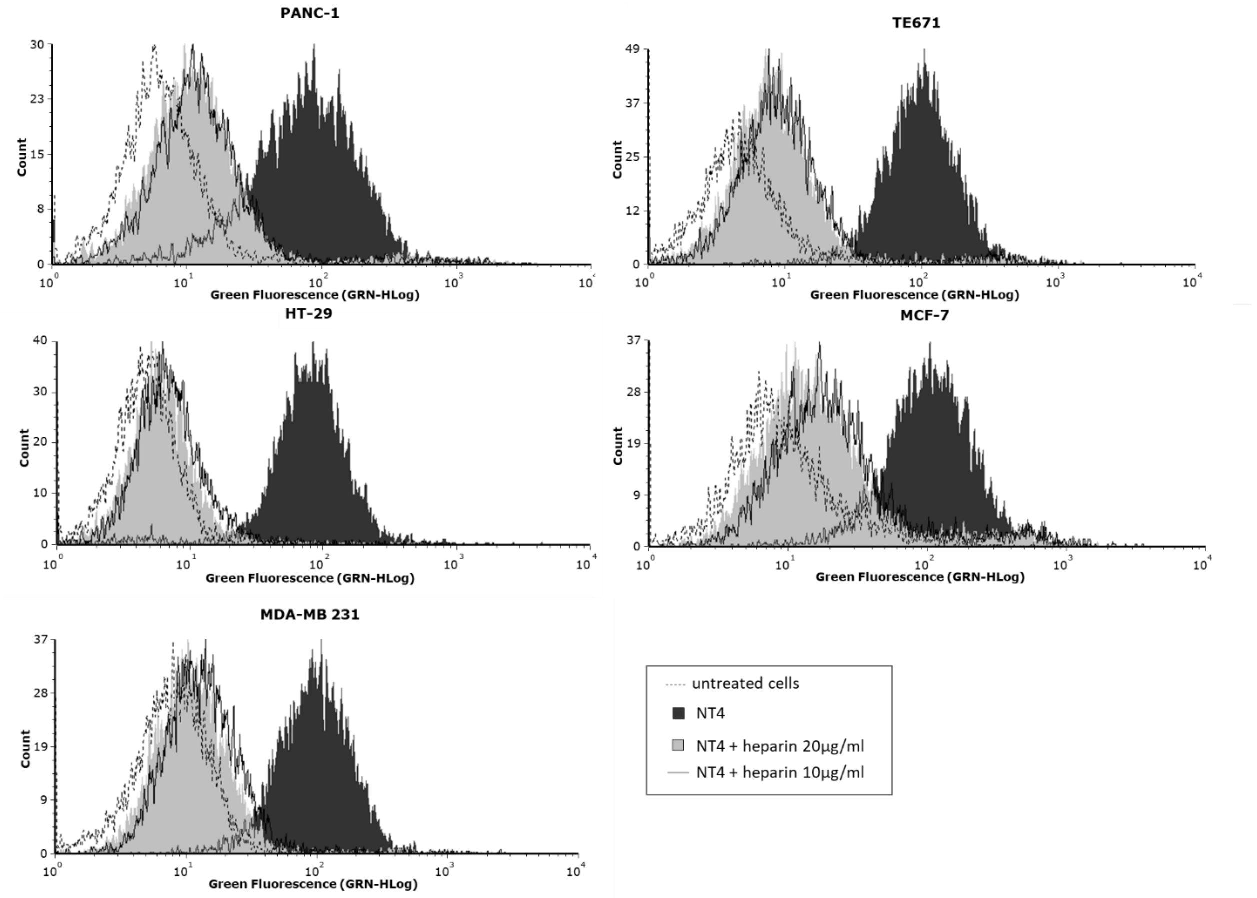


**Figure S1.** Flow cytometry analysis of NT4 specific binding (500 nM equivalent to 4 µg/ml) to PANC-1, TE671, HT-29, MCF-7 and MDA-MB-231 cancer cells in the presence of 20 or 10 µg/ml heparin.

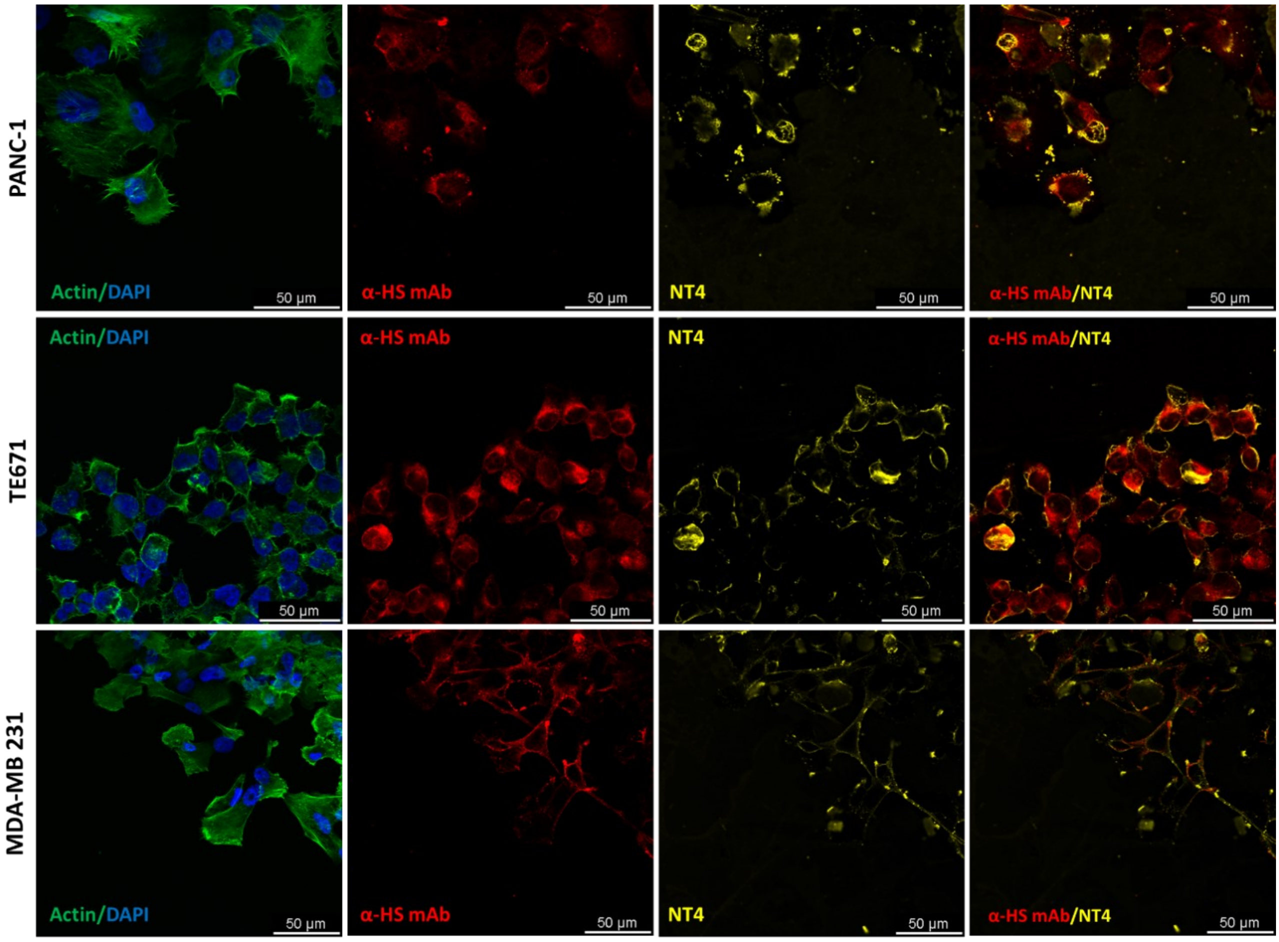


**Figure S2.** Co-localization of HSPGs in PANC-1, TE671 and MDA-MB 231 cell lines under migrating conditions. Confocal microscopy analysis of anti-HS 10E4 mAb (red), NT4 peptide (yellow), and co-staining (orange) in PANC-1, TE671 and HT29 cells. Nuclei are stained with 4',6-diamidino-2-phenylindole (DAPI; blue) and actin is stained with phalloidin (green).

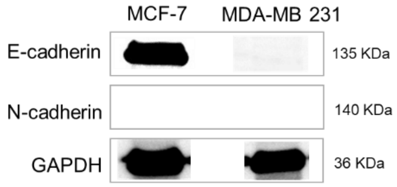


**Figure S3.** Expression of N- and E-cadherin by western blot in MCF-7 and MDA-MB 231 cells. GAPDH was tested as endogenous control.

**A**

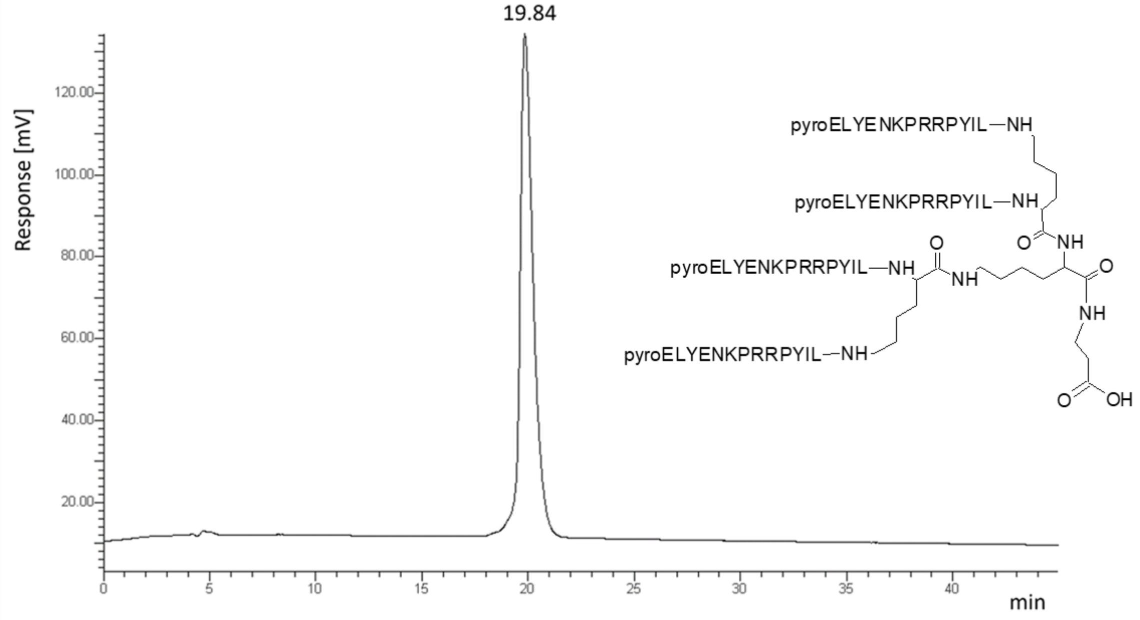


**B**

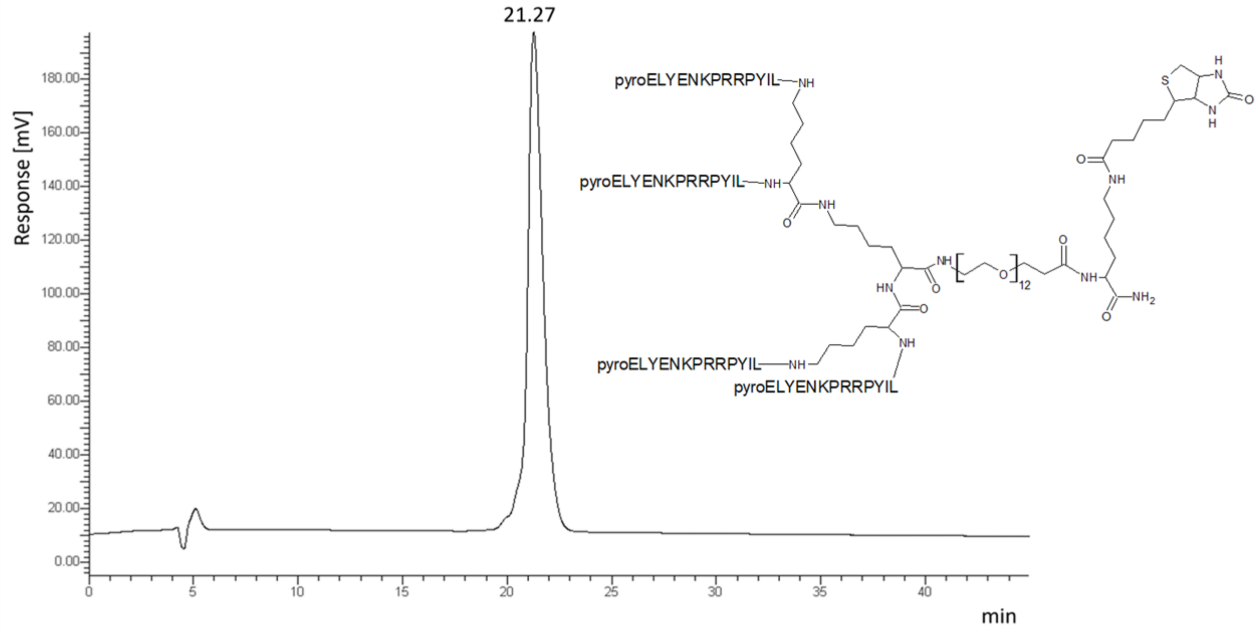


**Figure S4.** Structure and RP-HPLC profile after purification of tetrabranched NT4 peptide **(A)** and NT4 peptide conjugated with biotin **(B)**.

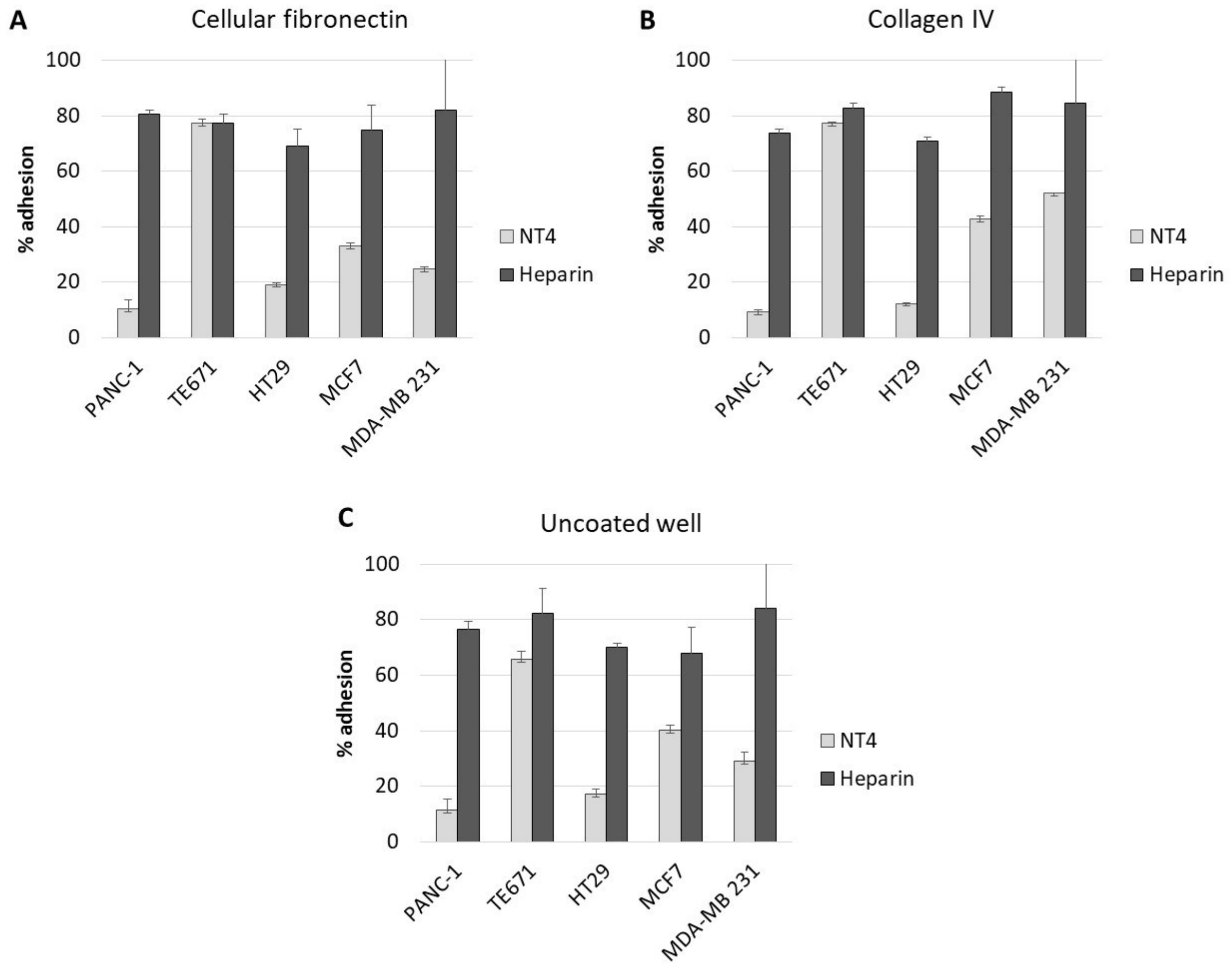


**Figure S5.** Percentage of cell adhesion after treatment with 70 µg/ml of NT4 and 70 µg/ml of heparin on cellular fibronectin (A), collagen IV (B) and uncoated well (C).
